# Effective Calibration of Low-Cost Soil Water Content Sensors

**DOI:** 10.3390/s17010208

**Published:** 2017-01-21

**Authors:** Heye Reemt Bogena, Johan Alexander Huisman, Bernd Schilling, Ansgar Weuthen, Harry Vereecken

**Affiliations:** Institute of Bio- and Geosciences, Agrosphere Institute (IBG-3), Forschungszentrum Jülich GmbH, 52425 Jülich, Germany; s.huisman@fz-juelich.de (J.A.H.); b.schilling@fz-juelich.de (B.S.); a.weuthen@fz-juelich.de (A.W.); h.vereecken@fz-juelich.de (H.V.)

**Keywords:** soil water content, permittivity, calibration, sensor-to-sensor variability, SMT100 sensor

## Abstract

Soil water content is a key variable for understanding and modelling ecohydrological processes. Low-cost electromagnetic sensors are increasingly being used to characterize the spatio-temporal dynamics of soil water content, despite the reduced accuracy of such sensors as compared to reference electromagnetic soil water content sensing methods such as time domain reflectometry. Here, we present an effective calibration method to improve the measurement accuracy of low-cost soil water content sensors taking the recently developed SMT100 sensor (Truebner GmbH, Neustadt, Germany) as an example. We calibrated the sensor output of more than 700 SMT100 sensors to permittivity using a standard procedure based on five reference media with a known apparent dielectric permittivity (1 < *K_a_* < 34.8). Our results showed that a sensor-specific calibration improved the accuracy of the calibration compared to single “universal” calibration. The associated additional effort in calibrating each sensor individually is relaxed by a dedicated calibration setup that enables the calibration of large numbers of sensors in limited time while minimizing errors in the calibration process.

## 1. Introduction

Knowledge of soil water content (SWC) is essential, as it represents a key variable in many hydrological, climatological, environmental and ecohydrological processes. In hydrology, SWC plays a major role in the water cycle by partitioning rainfall into runoff and infiltration [1], and by controlling hydrological fluxes such as interflow [2] and groundwater recharge [3]. SWC is also a key variable of the climate system, as it governs the energy fluxes between the land surface and the atmosphere through its impact on evapotranspiration [4]. Determining the temporal and spatial variability of SWC is hence essential for a wide range of studies, and a large number of measurement techniques have been developed in the past decades [5,6,7,8,9,10]. Recently, first initiatives started to compare different soil moisture sensors in the framework of calibration and validation sites for remotely sensed soil moisture [11].

Besides destructive gravimetric sampling, electromagnetic (EM) methods, such as time domain reflectometry (TDR) (e.g., [12]), time domain transmission (TDT) (e.g., [13]), and capacitance [14] and impedance sensors (e.g., [15,16]), are most commonly used for soil water content measurements at the point scale. All EM techniques rely on the dependency of the soil dielectric permittivity on the SWC. As the dielectric permittivity of liquid water is much higher than the dielectric permittivity of other soil components, SWC is the principal factor governing EM wave propagation in the soil. TDR and TDT sensors measure the propagation velocity of EM waves along open and closed transmission lines [13], respectively, whereas capacitance sensors typically determine SWC by measuring the charge time of a capacitor (i.e., the soil-probe system) for a given voltage [17]. Typically, capacitance sensors operate at a measurement frequency between 50 and 150 MHz, while TDR and TDT operate at higher frequencies. Higher frequencies are favorable because SWC measurements are expected to be less influenced by the electrical conductivity and the imaginary dielectric permittivity of the soil [18]. Nevertheless, the need for automated SWC measurement systems have largely increased the number of low-cost SWC sensors, e.g., for irrigation purposes [19].

More recently, wireless sensor networks have appeared as a promising approach to monitor SWC over large areas and with a high temporal resolution, which is particularly useful for observing ecohydrological processes [20,21], the spatial characterisation of soil properties (e.g., [22,23]) and for the validation of remotely sensed soil moisture products (e.g., [11]). Because of the multitude of SWC measurements within a sensor network, the interpretation of the sensor signal should be straightforward and unambiguous. Also, the SWC sensors need to be inexpensive in order to maximize the number of sensor nodes. Capacitance and TDT sensors are relatively inexpensive and easy to operate, and were found to be a promising choice for SWC measurements with wireless sensor networks (e.g., [24,25]). However, low-cost sensors can show considerable sensor-to-sensor variability (e.g., [26]), affecting the measurement accuracy if not appropriately accounted for. One possible solution would be a direct calibration between sensor response and soil water content for every sensor (e.g., [27,28,29]). However, several hundreds of sensors are used in the case of sensor network applications (e.g., [25,30,31]), which makes the direct calibration method impracticable.

Alternatively, a two-step calibration procedure (e.g., [32,33]) can be used. In a first step, the relationship between sensor response and permittivity is determined for each sensor (i.e., a sensor-specific calibration). In a second step, site-specific relationships between permittivity and SWC can be established with a limited number of measurements on soil samples, preferably using the highly accurate TDR method (soil specific calibration). For the sensor-specific calibration, media with well-known dielectric properties (here referred to as reference permittivity), such as air, 2-isopropoxyethanol [34] and 1,4-dioxane [35] are used to relate the sensor response to dielectric permittivity. The advantages of using this approach are: (i) the avoidance of air gaps and density variations; (ii) the possibility to separate sensor- and soil-specific effects; and (iii) the ability to quickly calibrate multiple sensors for a wide range of dielectric permittivity (from 2 to 35). In the second step, the dielectric permittivity is related to soil water content using empirical or semi-empirical models (e.g., [36,37]). For more accurate SWC measurements, a site-specific calibration accounting for soil textural variation can also be performed on a limited number of samples (e.g., [30,31]).

The objectives of this study are (i) to introduce the recently developed low-cost SMT100 SWC sensor; (ii) to present an effective calibration method for low-cost EM sensors taking the SMT100 as an example and (iii) to demonstrate the increase in accuracy when a sensor-specific calibration is used instead of a single “universal” calibration between sensor response and dielectric permittivity.

## 2. Materials and Methods

### 2.1. The SMT100 Soil Water Content Sensor

**Figure 1:**
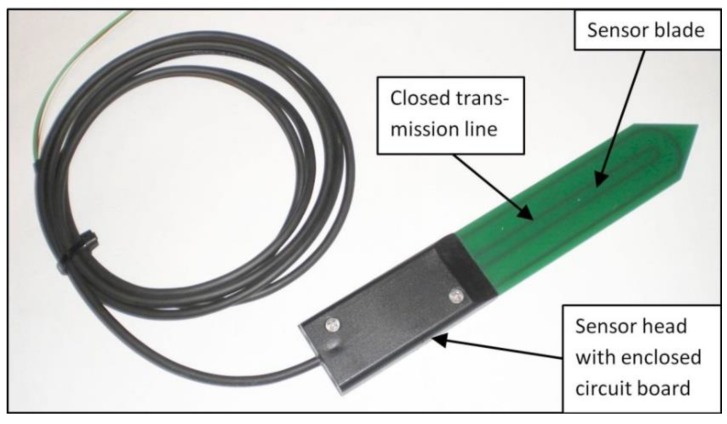
The SMT100 soil water content sensor.

The SMT100 soil water content sensor is the successor of the SPADE sensor [25]. Both sensors use a ring oscillator in which a steep pulse (<300 ps pulse rise time), emitted by a line driver, travels along a closed transmission line buried in the soil. The closed transmission line consists of two copper strips embedded in a circuit board, which is 30 mm wide and 120 mm long (Figure 1). The sensor head that contains the sensor electronics is 80 mm long. Instead of measuring the pulse travel time directly as TDT sensors do, the pulse is inverted and then fed back to the input of the line driver. This results in an “oscillation” frequency that mainly depends on the pulse travel time and thus the soil dielectric permittivity (see [25]) for a more detailed technical description). The oscillation frequency is approximately 150 MHz in water and 340 Hz in air. In addition to soil water content, the SMT100 sensor measures temperature using a digital temperature sensor (ADT7410, Analog Devices Inc., Norwood, MA, USA) with an accuracy of ±0.4 °C from −10 °C to 85 °C. The SMT100 type used in this study is equipped with a digital interface (SDI-12) (Truebner GmbH, Neustadt, Germany) that ensures minimal interference by electromagnetic noise. The SMT100 uses a more ruggedized jacket compared to the SPADE sensor to ensure a longer life time of the sensor even for harsh soil conditions (frozen soil conditions, low pH, etc.). For this study, we used a customized version of the SMT100 sensor optimized for wireless sensor network applications with very low power consumption. The power requirement of this customized SMT100 sensor is very low with about 50 mA during the measurement time of about 50 ms. Additionally, new SDI-12 commands have been implemented to enable sensor-specific calibration of the SMT100 sensor by storing the individual calibration parameters. For this study, we used a batch of 701 SMT100 sensors, which were produced for a wireless sensor network in the Rollesbroich headwater catchment in Germany [31].

We have performed a temperature experiment with three SMT100 sensors (between 4 °C and 20 °C) to test whether the sensor electronics of the SMT100 sensor show any sensitivity to temperature changes. For this test, we used the experimental setup described in [25]. The soil was a silty loam sample taken from the TERENO test site Selhausen in Germany [38]. The apparent dielectric permittivity was modeled using the Complex Refraction Index Model (CRIM, [39]):

(1) $$\theta =100\times \frac{K_{a}^{\beta }-\left(1-\eta \right)\times K_{s}^{\beta }-\eta K_{air}^{\beta }}{K_{water}{\left(T\right)}^{\beta }-K_{air}^{\beta }}$$

where *ƞ* is the porosity of the soil, *β* is a shape factor which is assumed to be 0.5, *K_a_* is the measured apparent dielectric permittivity, and *K_water_*, *K_solid_*, and *K_air_* are the permittivity of water, solids, and air, respectively. The permittivity of *K_solid_* and the soil porosity were assumed to be 3.5 and 0.38, respectively. The dielectric permittivity of air (*K_air_*) is 1, and that of water (*K_water_*) is a function of temperature *T*, and can be calculated by [40]:

(2) $$K_{water}=78.54\times \left[1-4.579\times 10^{-3}\left(T-25\right)+1.19\times 10^{-5}{\left(T-25\right)}^{2}-2.8\times 10^{-8}{\left(T-25\right)}^{2}\right]$$

With Equations (1) and (2) we can eliminate the temperature sensitivity effect of the dielectric permittivity of water on the sensor reading. Any remaining temperature effect should be related to the temperature sensitivity of the sensor electronics [25].

### 2.2. Calibration Standards

Liquids like organic solvents are often used as reference media for SWC sensor calibration as they are easily available and enable a good contact with the sensor prongs without air-gaps (e.g., [13,41]). In order to increase the range of possible dielectric permittivity values used in calibration, the permittivity of pure organic solvents can be adjusted by the addition of water, or solids such as glass beads [42]. However, such mixtures may introduce uncertainty due to demixing of liquids or variations in bulk density in glass beads packings. In addition, reference liquids may alter during the calibration process, e.g., by hygroscopic adsorption of water vapour, partial degassing, or contamination. Therefore, the status of the liquids should be checked from time to time to ensure repeatability of the sensor calibration procedure.

In this study, five calibration standards were used for sensor calibration (air, glass beads and three mixtures of 2-isopropoxyethanol (i-C3E1) and deionised water with a defined volume fraction of i-C3E1) (Table 1). The permittivity of the five reference media ranges from 1 (air) to 34.8 covering most of the dielectric permittivity values found in natural soils. The glass beads are soda lime glass beads (type: Silibeads 4501, Sigmund Lindner GmBH, Germany) with a grain size of 0.25–0.5 mm, which consist of 72.5% SiO_2_, 13% Na_2_O, 9.06% CaO, 4.22% MgO and 0.58% Al_2_O_3_. [43] reported that packings of these glass beads have a static permittivity of 3.34 at 25 °C at the maximum achievable package density that resulted in a porosity of 38 vol.%. (Table 1). They used a PNA E8363B network analyser (Agilent Technologies, Ratingen, Germany) in combination with a 7-16-100 coaxial transmission line (Rosenberger, Fridolfing, Germany). The frequency-dependent complex dielectric permittivity of the three i-C3E1/water mixtures (M3 to M5) was measured at 25 °C in a frequency range from 0.5 to 10 GHz using a dielectric probe kit with a slim probe (Agilent 85070E, Agilent Technologies Inc., Santa Clara, CA, USA) and a network analyzer (HP 8720A, Agilent Technologies Inc., Santa Clara, CA, USA) by [44]. The volume fractions and the reference dielectric permittivity of the three reference liquids (M3–M5) are also listed in Table 1. By using dry glass beads and mixtures of deionized water and 2-isopropoxyethanol with negligible electrical conductivity, we ensured that the sensor calibrations were not influenced by electrical conductivity effects.

### 2.3. Calibration Setup

**Figure 2:**
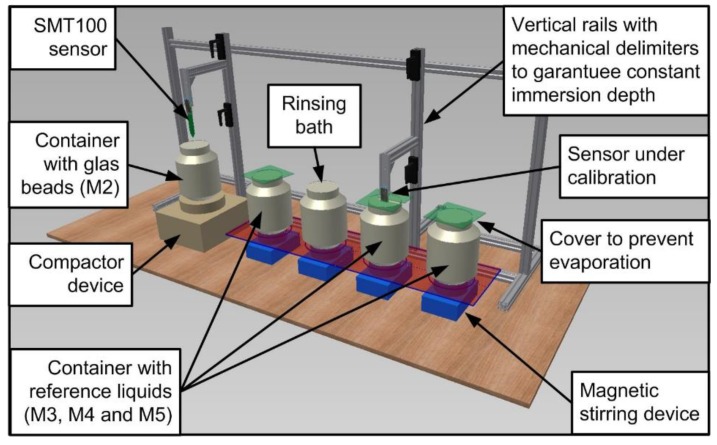
Schematic drawing of the calibration station.

**Figure 3:**
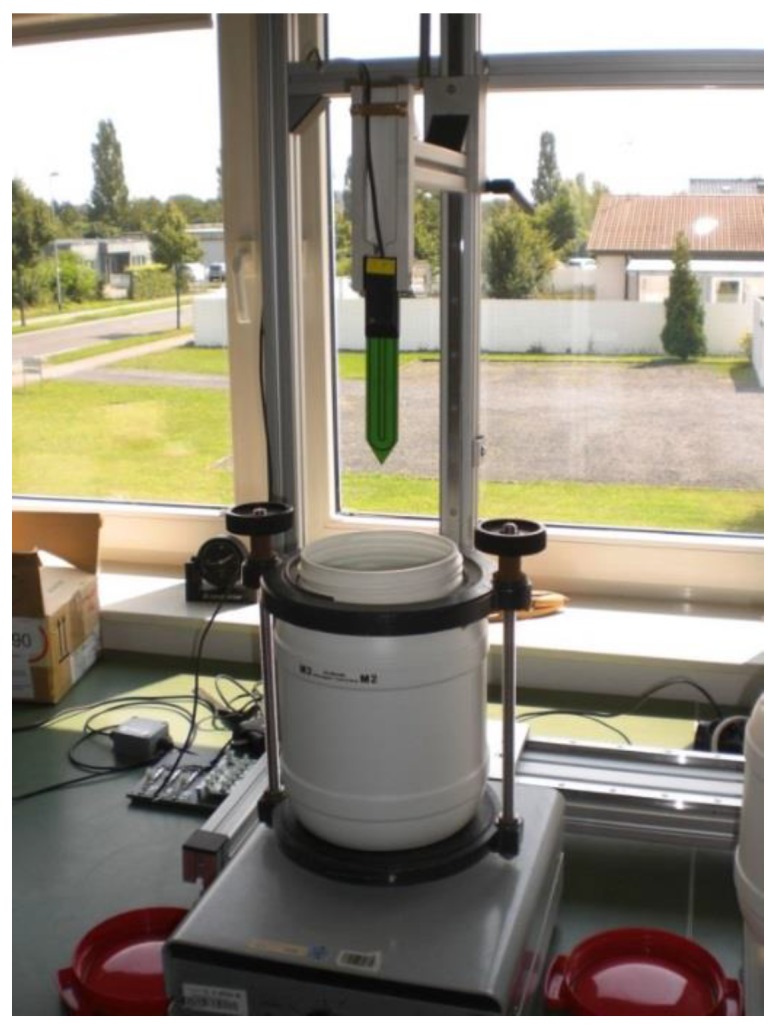
The container with glass beads (M2) on top of the vibration machine.

In order to obtain precise measurements during sensor calibration, it is important to take several precautions [44]. First, the sensors need to be completely immersed in the standard liquid, which requires a sufficiently dimensioned container. Here, we used 6.4 dm^3^ bottles (diameter of 19.5 cm, height of 23.0 cm, high density polyethylene) to ensure that the area of influence of the SMT100 sensor is completely captured. Second, the sensor needs to be fixed and centrally immersed into the bottle to reduce effects of positioning on the measurements. Finally, possible degrading effects of standard liquids on the sensor plastic body need to be minimized by carefully cleaning the sensor after each measurement and minimizing the contact time. In order to guarantee that these precautions were met in the best possible way and to allow calibration of a large number of sensors in a limited amount of time, a dedicated calibration station was developed and manufactured at Forschungszentrum Jülich GmbH (Figure 2). The calibration station consists of five containers arranged in parallel on a work bench. The density of the glass beads packing (M2) affects the reference permittivity value of this calibration medium. The reference permittivity value used in this study corresponds to the maximum density of the glass beads packing according to [43]. However, insertion and removal of the SMT100 sensor during the calibration process may affect the density of the glass beads packing. In order to ensure that the density of the packing remains optimal during calibration, a vibration machine is used to compact the glass beads packing to the maximum density before each calibration measurement (see Figure 3). In order determine the time needed for achieving the maximum density of the glass beads packing after sensor insertion, we conducted a compaction experiment with the vibration machine (see Section 3.2). For each experiment, a standard SMT100 sensor was fully inserted into the glass beads and the sensor response was recorded every 15 s for a time period of 300 s.

The other containers are filled with mixtures of 2-isopropoxyethanol (i-C3E1) and deionized water (M3–M5). These containers are placed on magnetic stirring devices to avoid demixing of the reference liquids. In addition, they are covered with a lid to prevent evaporation. The reference liquids are tested from time to time using reference SWC sensors to check whether the reference permittivity value has not changed due to evaporation or contamination. One additional container is used to clean the sensors after each measurement (denoted as rinsing bath). A framework behind the containers holds two vertical rails on which the SMT100 sensors can be fixed and moved upwards and downwards (Figure 2). Mechanical delimiters on the vertical rails guarantee a predefined immersion depth of the SMT100 sensors. The calibration station is designed in such a way that two persons can work in parallel.

### 2.4. Relating Sensor Output to Dielectric Permittivity

The measured oscillation frequency by the SMT100 sensor has to be converted to an apparent dielectric permittivity, *K_a_*. In principle, the sensor response could be modelled using an appropriate electrical circuit model. Unfortunately, such a model is not yet available. Therefore, the sensor response (counts) is related to *K_a_* using the following empirical model:

(3) Ka=γ+1α+β(18000−counts)5000

where *α*, *β*, and *γ* are fitting parameters (Table 2 lists the parameter values). The root mean square error (RMSE) between the predicted *K_a_* and the known reference permittivity was used to quantify the accuracy of the empirical functions. In addition, we present the calibration results in terms of equivalent SWC calculated using the polynomial empirical permittivity SWC relationship of [36]. This information is provided to allow a better understanding from the hydrological point of view and does not imply a direct calibration between sensor response and soil water content.

## 3. Results and Discussion

### 3.1. Temperature Effect of the SMT100 Sensor

**Figure 4:**
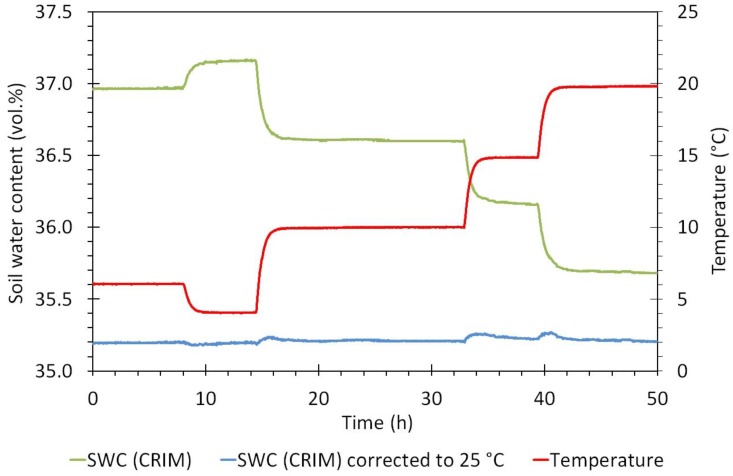
The average temperature and soil water content response of three SMT100 sensors. Soil water content was derived from measured apparent permittivity using the complex refraction index model (CRIM) and the temperature correction was done using the temperature dependence of the dielectric permittivity of water reported by [43].

The average temperature and soil water content response of three SMT100 sensors is presented in Figure 4. Clearly, temperature has a significant influence on the sensor output of the SMT100 sensor. However, after correcting the sensor response using the temperature dependence of the dielectric permittivity of water from [40], the temperature sensitivity disappeared to a large extent. Only some minor fluctuations are present in transient periods the range within the corrected permittivity estimate varies (~0.1 vol.%) due to a time delay between the equilibration of the temperature sensor and the entire sensor electronics. This indicates that change in permittivity with changing temperature is solely related to the temperature sensitivity of the dielectric permittivity of water and that the sensor electronics are showing no temperature effect. Since the SMT100 sensor measures temperature, users can easily apply our correction approach.

### 3.2. Density of the Glass Beads Packing

**Figure 5:**
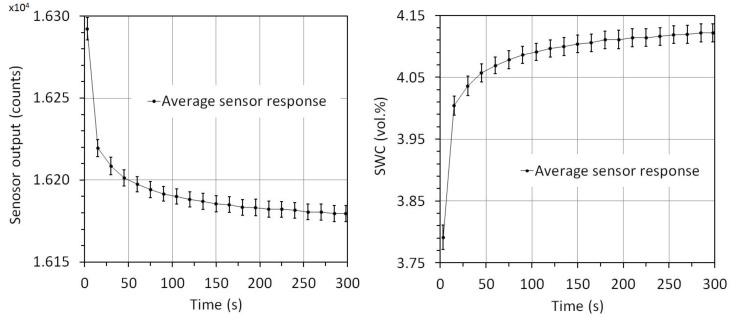
Results of the compaction experiment with glass beads packing (left and right panels present the raw sensor output and equivalent SWC calculated with the Topp equation [36], respectively).

Figure 5 shows the decrease in sensor response due to the increasing density of the glass beads packing during the compaction experiment. Clearly, the strongest changes in sensor response occurred in the first 15 s. Subsequently, the change in sensor response continuously decreased until the end of the experiment (after 300 s). The total difference in terms of raw counts was 113, which corresponds to an increase of equivalent SWC of 0.33 vol.% (Figure 5). Based on these results, it was decided to perform the calibration measurement after 120 s as a compromise between calibration efficiency and accuracy. The change in sensor response due to further compaction from 120 to 180 s was negligible with approx. 0.05 vol.%.

### 3.3. Alteration of the Reference Liquids

**Figure 6:**
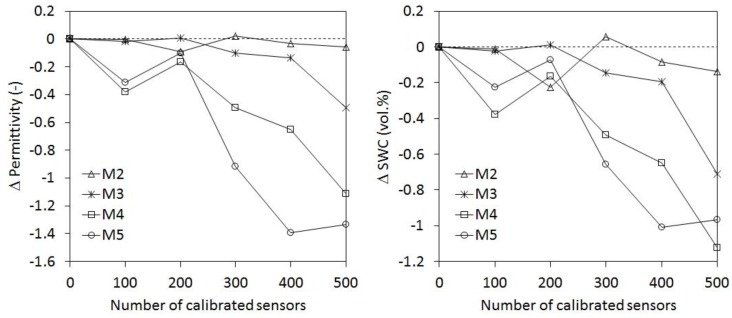
Alteration of reference liquids (M3–M5) during the calibration of 500 SMT100 sensors (left and right panel present permittivity and equivalent SWC calculated with the Topp equation [36], respectively). The values represent the deviations from the permittivity of the fresh reference media. The deviations were derived from the average response of two SMT100 sensors that were repeatedly used to measure the permittivity during the calibration of 500 SMT100 sensors (i.e., always after 100 calibrations). The results for M2 (glass beads) are also presented for comparison.

In order to ensure repeatability of the sensor calibration procedure, we evaluated changes in the permittivity of the reference liquids during calibration of a large amount of sensors. In this experiment, two SMT100 sensors were repeatedly used to measure the permittivity of the reference media during the calibration of 500 SMT100 sensors. Each measurement was repeated once, which resulted in four measurements in each reference media at a particular time. The whole experiment took 14 days. Figure 6 presents the arithmetic mean of the measured reference permittivity obtained using a sensor-specific calibration using the fresh reference media. In order to better visualize changes in the permittivity of the reference media, we plotted here the deviation from the permittivity of the fresh reference media. Figure 6 shows that the permittivity of the reference liquids did not vary strongly during the calibration of the first 200 sensors. The variation in measured permittivity is in the range of the accuracy of the SMT100 sensor (see next section). However, the permittivity of all reference liquids started to decrease after 300 calibrations. On average, the permittivity decreased by 3.6% between 200 and 500 calibrated sensors, which corresponds to a mean SWC decrease of 0.89 vol.%. Clearly, the alteration of the reference liquids cannot be explained with evaporation effects of the more volatile i-C3E1, because this would have led to an increase in permittivity. Also, a cleaning effect (entry of cleaning water into the reference media) is unlikely, because this would led to higher permittivity values of the reference media. The reference permittivity of M2 (glass beads) does not show a decrease (Figure 6), which suggest that the overall procedure is repeatable and that the observed deviations in permittivity must be attributed to changes within the liquids. Although the reason for the observed decrease remains unclear, as a result of this experiment we recommend to closely monitor the properties of the reference media using the analysis approach used here and to change liquid reference media after the calibration of about 200 SWC sensors.

### 3.4. Sensor-to-Sensor Variability

**Figure 7:**
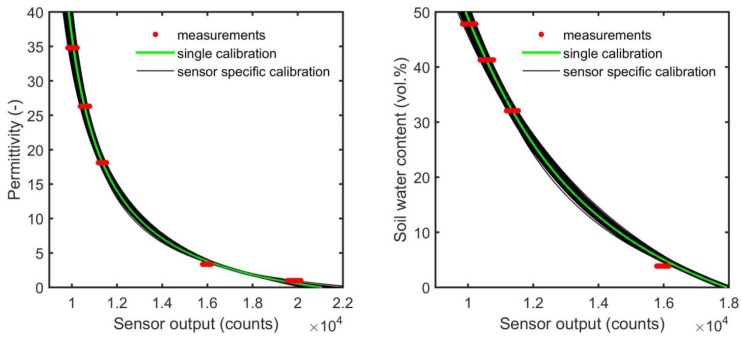
Sensor-specific calibration curves fitted to the sensor response measurements of each of the 701 SMT100 sensors, as well as the single calibration curve fitted to the whole data set of every sensor type (left and right panels are presenting permittivity and equivalent soil water content values, respectively).

Figure 7 and Table 3 illustrate the sensor-specific calibration results of the 701 SMT100 sensors. The observed sensor-to-sensor variability visible in Figure 7 and Table 3 is a consequence of intrinsic factors, such as variations in the electrical components affecting propagation delay and pulse rise/fall time and tiny variations in the probe geometry, e.g., the circuit board which embeds the transmission line [25].

The calibration parameters of the single calibration model as well as the statistics from all 701 sensor calibrations are presented in Table 2. For the 701 sensor calibrations, the average and worst correlation coefficients were 0.9997 and 0.9946, respectively. This indicates that the calibration function was able to fit the data extremely well. One way to further increase the reliability of the calibration procedure would be to use an additional reference medium between M2 and M3. For instance, mixtures of Dioxane and water could be used (e.g., [17]). However, Dioxane is carcinogenic and thus not desirable to be used for mass calibration of SWC sensors [42]. In future studies, we will investigate whether other reference liquids (e.g., Dimethylsulphoxide, Quinoxaline) are similarly or even better suitable for the SWC sensor calibration procedure.

### 3.5. Sensor-Specific versus Single Calibration

For wireless network application in which a very large number of SWC sensor would need to be calibrated, it is of interest to determine the decrease in accuracy when sensor-to-sensor variability is ignored. Therefore, Figure 7 also presents a single “universal” calibration curve which was derived from the complete set of calibration measurements. The RMSE (root mean square error) between reference permittivity and apparent permittivity estimated using the calibration model (Equation (1)) can be used as a measure of accuracy in this study (Table 4). It should be noted that the RMSE reported here does not include errors in the reference permittivity.

For the single calibration, the RMSE seems to increase slightly with permittivity (Table 4). The overall RMSE for the universal calibration was ~0.87 (~0.95 vol.%) for the permittivity range from 3.34 to 34.8. This is already a relatively low RMSE value indicating that sensor-to-sensor variability is moderate and that accurate SWC measurements are also possible with the SMT100 sensor without accomplishing a sensor-specific calibration. However, when this sensor-to-sensor variability was removed by sensor-specific calibration, the RMSE for this permittivity range decreased to ~0.27 (~0.48 vol.%). [44,45] found similar or higher errors related to sensor-to-sensor variability for the popular low-cost SWC sensors EC-5 and 5TE (Decagon Devices). According to [44], RMSE values of these sensors were 1.5 (1.6 vol.%) and 1.2 (1.0 vol.%), respectively. However, it has to be noted that they used different reference media for calibration, which might have an effect on the RMSE values. Similar to the results presented here, [44] also reported a substantial decrease in measurement error if sensor-specific calibration is used (e.g., the RMSE decreased by about 50% for the EC-5 sensor).

## 4. Conclusions

In this paper, we present an effective calibration procedure for electromagnetic soil water content sensors taking the low-cost SMT100 sensor as an example. We calibrated the sensor output of 701 SMT100 sensors to permittivity using a set of reference media with known apparent dielectric permittivity in the range between 1 and 34.8. We investigated the properties of the reference liquids during the calibration process and found strong alterations after the calibration of about 200 SWC sensors. We therefore recommend to carefully check the properties of reference media every 100 measurements. Furthermore, we compared the accuracy of a single universal calibration with the accuracy that can be achieved using sensor-specific calibration. Our results showed that a sensor-specific calibration strongly improved the accuracy of the calibration compared to single calibration, decreasing the RMSE by about 70% (from 0.87 to 0.27). The associated additional effort in calibrating each sensor individually is relaxed by a dedicated calibration setup that enables the calibration of large numbers of sensors in a limited time while minimizing errors in the calibration process.

## Figures and Tables

**Table 1:** The reference permittivity of the calibration media as well as the equivalent soil water content (SWC) calculated with the Topp equation [36].

Calibration Standard	Medium	Reference Permittivity	Volume Fraction i-C3E1	Equivalent SWC
		(-)	(-)	(Vol.%)
M1	Air	1	-	-
M2	Glass beads	3.34	-	0.386
M3	i-C3E1/water mixture	18.1	0.92	32.1
M4	i-C3E1/water mixture	26.3	0.8	41.1
M5	i-C3E1/water mixture	34.8	0.68	47.8

**Table 2:** Calibration parameters of the single calibration model as well as the statistics from all 701 sensor calibrations.

Parameter	Single Calibration	Minimum	Maximum	Standard Deviation
*α*	−0.1305	−0.1689	−0.1013	0.0047
*β*	0.2549	0.2110	0.3123	0.0072
*γ*	1.8342	1.6594	1.9960	0.0470

**Table 3:** Statistical results of the sensor response measurements using 701 SMT100 sensors.

Calibration Standard	Mean Sensor Response	Standard Deviation	Coefficient of Variation
	(Counts)	(Counts)	(%)
M1	19,816	79.1	0.40
M2	15,969	55.7	0.35
M3	11,386	58.5	0.51
M4	10,559	66.1	0.63
M5	10,072	70.0	0.69

**Table 4:** RMSE between measured and reference permittivity using sensor-specific calibration and a single universal calibration, respectively, as well as the corresponding equivalent SWC calculated with the Topp equation [36].

Calibration Standard	RMSE Sensor-Specific Calibration		RMSE Single Calibration Function	
	Permittivity (-)	Equivalent SWC (Vol.%)	Permittivity (-)	Equivalent SWC (Vol.%)
M1	0.373	-	0.370	-
M2	0.511	1.298	0.511	1.390
M3	0.258	0.350	0.483	0.660
M4	0.229	0.213	0.920	0.830
M5	0.084	0.055	1.565	1.012
